# Feasibility of omitting the unenhanced phase in CT angiography for acute aortic pathologies

**DOI:** 10.1371/journal.pone.0362760

**Published:** 2026-11-13

**Authors:** Soo Buem Cho, Jieun Byun

**Affiliations:** Department of Radiology, College of Medicine, Ewha Womans University, Seoul, Republic of Korea; Universitaria di Bologna, ITALY

## Abstract

**Objectives:**

To evaluate the feasibility of a contrast-only computed tomography angiography (CTA) protocol versus a conventional multiphase protocol for detecting and characterizing acute aortic pathologies, and reducing radiation dose.

**Materials & methods:**

This retrospective study included 160 patients who underwent multiphase CTA for evaluating acute aortic syndrome. Two readers independently evaluated 640 predefined aortic segments for the presence of aneurysms, dissections, intramural hematomas (IMH), and penetrating atherosclerotic ulcers (PAU). Non-inferiority was assessed using generalized estimating equations with a prespecified 5% margin. Inter-protocol and inter-reader agreements were calculated using Cohen’s kappa. Radiation dose also was compared**.**

**Results:**

The contrast-only protocol demonstrated non-inferiority to the full protocol for detecting all major aortic pathologies, with all confidence intervals falling within the 5% margin. Detection rates were identical for aneurysms (33.6% vs. 33.6%; *p* = 1.000) and similar for dissections (15.6% vs. 15.9%; *p* = 0.500). Detection rates were slightly higher with the contrast-only protocol for IMH (5.8% vs. 5.0%) and PAU (5.8% vs. 4.7%), primarily reflecting false-positive interpretation. Interprotocol agreement was perfect for aneurysm shape and rupture status (kappa = 1.000) but lower for mural thrombus characterization (kappa = 0.703). The contrast-only protocol achieved a 32.1 ± 5.7% reduction in mean DLP and 32.4 ± 4.5% for CTDI vol (both, *p* < 0.001).

**Conclusion:**

In patients with acute aortic pathology, a contrast-only protocol demonstrated non-inferior diagnostic performance to the full multiphase protocol, with comparable reader agreement and a 32% reduction in radiation dose. These findings suggest that omitting the unenhanced phase may be feasible in selected clinical scenarios, particularly in patients undergoing serial surveillance imaging, where cumulative dose reduction is a priority.

## Introduction

Acute aortic pathology encompasses a spectrum of life-threatening conditions, including classic acute aortic syndrome—such as aortic dissection, intramural hematoma (IMH), and penetrating atherosclerotic ulcer (PAU)—as well as acute complications of aortic aneurysms, which share similar clinical presentations but require distinct management strategies [1,2]. Computed tomography angiography (CTA) is the gold standard for the rapid and accurate diagnosis of acute aortic pathology [3]. Although protocols vary across institutions, a standard acquisition typically includes an unenhanced phase followed by an arterial phase and delayed phase. The unenhanced phase is traditionally regarded as essential, particularly for detecting IMH, as the high-attenuation crescent of the hematoma can be obscured by luminal contrast enhancement or mimicked by atherosclerotic plaque in the post enhanced images [4, 5].

However, the inclusion of an unenhanced phase inevitably increases the radiation burden and prolongs the examination time [6]. This is of particular concern given the rising cumulative radiation exposure in patients with aortic disease who require lifelong surveillance imaging [7]. Furthermore, the necessity of the unenhanced phase has been increasingly questioned in the era of modern multidetector CT scanners. High-spatial-resolution imaging may allow for the detection of IMH and other subtle pathologies based solely on morphological changes, such as focal intimal displacement or wall thickening, even without unenhanced baseline images [8–10].

While previous studies have evaluated reduced-dose protocols, data specifically confirming the non-inferiority of a contrast-only protocol for the full spectrum of acute aortic pathology —especially for subtle entities like PAU and IMH—remain limited. Establishing whether the unenhanced phase can be safely omitted in patients with confirmed aortic pathology—without compromising diagnostic performance—could offer meaningful radiation dose benefits, particularly for patients requiring serial imaging.

Therefore, the purpose of this study was to assess the diagnostic non-inferiority of a contrast-only CTA protocol compared with a standard full multiphase protocol for the detection of acute aortic pathologies and to quantify the potential radiation dose reduction.

## Materials and methods

### Study population

This retrospective study was approved by the Institutional Review Board of Ewha Womans University Seoul Hospital, which waived the requirement for written informed consent because of the retrospective nature of the study. The study was conducted in accordance with the principles of the Declaration of Helsinki. 615 Consecutive patients who underwent CTA for suspected acute aortic pathology between Jan.2020 and Dec. 2024 were identified. Data were accessed for research purposes between May 28, 2025, and Apr. 30, 2026. The authors did not have access to information that could identify individual participants during or after data collection. Patients were included if their final diagnosis was confirmed as aneurysm, classic aortic dissection, intramural hematoma (IMH), or penetrating atherosclerotic ulcer (PAU), and if both unenhanced CT and contrast-enhanced phases were available. Exclusion criteria were: (a) nondiagnostic image quality, (b) markedly altered postoperative aortic anatomy. Finally, 160 patients were included in this study.

### CTA acquisition and image preparation

All CT scans were performed on 3 different scanners: SOMATOM Force (Siemens Healthineer, Erlangen, Germany), Revolution Frontier CT and Revolution CT (GE Healthcare, Waukesha, WI). CT acquisition parameters are summarized in S1 Table. All patients underwent multiphase protocol, consisting unenhanced, arterial, and delayed phases. The unenhanced scans were performed first, then 120 mL of contrast medium (iohexol 350 mg I/mL; 600 mg I/kg based on a 70 kg standard) was injected intravenously at a rate of 4 mL/s using an automatic power injector. For arterial phase, a bolus triggering algorithm was used, which automatically started the scan 3 s after a prespecified threshold of 150 HU was reached in the descending thoracic aorta. The delayed phases were obtained 180 s after the administration of the contrast medium. All phases utilized a window range from the upper level of the thyroid to the great trochanter. Reconstruction was carried out using a slice thickness of 2.0 mm and without any interslice gap for both the unenhanced and contrast-enhanced images.

### Image evaluation

All CT examinations were reviewed independently by two experienced radiologists (J.B. and S.B.C) with over 15 years of interpretating CTA. For each patient, the thoracic and abdominal aorta were divided into predefined anatomical segments -ascending aorta, aortic arch, descending thoracic aorta, and abdominal aorta- and the readers evaluated the presence or absence of the four major aortic pathologies-aneurysm, dissection, IMH, and PAU-within each segment. Aortic aneurysm was defined according to segment-specific diameter thresholds: ≥ 5.0 cm for the ascending aorta, ≥ 4.0 cm for the aortic arch, and ≥ 3.0 cm for the descending aorta [11,12]. For segments with an identified aneurysm, the readers further evaluated detailed morphological characteristics including macroscopic shape, mural thrombus pattern, intimal calcification, draped aorta sign, and rupture status [13–18]. Predefined CT criteria was summarized in S2 Table. In the first session, only contrast-enhanced datasets were interpreted. After a 4-week interval to reduce recall bias, the readers evaluated the full-protocol datasets, which included both unenhanced and contrast-enhanced images. Interpretations from the two sessions were recorded independently, and any discrepancies between readers were resolved by consensus review.

Volume CT dose index (CTDI vol) and dose length product (DLP) values of the CTA were extracted for all examinations.

### Statistical analysis

Detection rates for aortic pathologies were compared between the contrast-only and full protocols using McNemar’s exact test, with corresponding exact 95% confidence intervals calculated for the paired proportion differences. Non-inferiority of the contrast-only protocol relative to the full protocol was assessed separately using generalized estimating equation (GEE) models with an identity link and an exchangeable correlation structure to account for within-patient clustering arising from multiple aortic segments per patient. Absolute differences in detection proportions (contrast-only minus full protocol) and their model-derived 95% confidence intervals were estimated. A prespecified non-inferiority margin of 5% (Δ = −0.05) was used, and non-inferiority was declared if the lower bound of the model-derived 95% CI for did not exceed −0.05 [19,20]. Interprotocol agreement for aneurysm characterization was evaluated using Cohen’s kappa coefficients. Inter-reader agreement for each protocol agreement between readers were also assessed using kappa statistics with 95% confidence intervals. To compare radiation dose parameters between full protocol and contrast only protocol, a paired t-test was performed for DLP and CTDI vol. All statistical tests were two-sided, and a *p*-value < 0.05 was considered statistically significant. All statistical analyses were performed using R (version 4.5.2) and SPSS (version 22; IBM Corp).

## Results

A total of 160 patients (mean age, 69.7 ± 14.6 years; 117 men [73.1%]) were included in the analysis. Baseline demographic and clinical characteristics are summarized in Table 1. The contrast-only protocol resulted in a significantly lower radiation dose compared with the full protocol. The mean DLP decreased from 1392.4 ± 706.7 mGy·cm to 954.0 ± 500.9 mGy·cm (*p* < 0.001), and the mean CTDI vol decreased from 18.6 ± 9.2 mGy to 12.7 ± 6.6 mGy (*p* < 0.001). This corresponded to a mean radiation dose reduction of 32.1 ± 5.7% for DLP and 32.4 ± 4.5% for CTDIvol.

**Table 1:** Baseline demographic and clinical characteristics (n = 160).

Characteristic	Value
**Age (years, Mean ± SD)**	69.7 ± 14.6
**Sex (n,%)**	
**Male (n,%)**	117 (73.1)
**Female**	43 (26.9)
**SBP/DBP (mmHg, Median (IQR))**	133 (121–150)/ 75 (63–84)

SD, standard deviation; IQR, interquartile range; SBP, systolic blood pressure; DBP, diastolic blood pressure

### Detection and characterization of aortic pathologies

Across 640 aortic segments obtained from 160 patients, the detection rates of major aortic pathologies-including aneurysm, dissection, IMH, and PAU-were highly comparable between the contrast-only and full protocols (Table 2). Detection of aortic aneurysm was identical in both protocols (215 segments, 33.6%), with no discordant cases (*p* = 1.000). For aortic dissection, the full protocol yielded a marginally higher detection rate (15.9% vs. 15.6%); however, this difference was not statistically significant (*p* = 0.500). IMH and PAU detection showed higher detection rates with the contrast-only protocol (5.8% vs. 5.0% and 5.8% vs. 4.7%, respectively), although the difference did not reach statistical significance (*p* = 0.180 and 0.065, Fig 1). All observed detection differences (Δ) were small in magnitude, and the 95% confidence intervals for each comparison remained entirely within the prespecified non-inferiority margin of 5%. A sensitivity analysis using GEE models to account for within-patient clustering yielded qualitatively similar results, with all 95% Cis remaining within the non-inferiority margin (S4 Table). Table 3 demonstrated the diagnostic performance of the contrast-only protocol for major aortic pathologies using the full protocol as the reference standard. The contrast-only protocol showed high sensitivity (93.3–100%) and specificity (98.5–100%) across all pathologies. While no discrepancies were observed for aneurysm, dissection showed two false-negative cases, whereas IMH and PAU demonstrated a small number of false-positive cases (Fig 2).

**Table 2:** Detection rates of acute aortic pathologies using contrast-only and full CT protocols (n = 640).

Pathology	Contrast-only, n (%)	Full protocol, n (%)	p-value	Difference in detection proportion (95% CI)
**Aneurysm**	215 (33.6)	215 (33.6)	1.000	0.000 (0.000–0.000)
**Dissection**	100 (15.6)	102 (15.9)	0.500	−0.003 (−0.003–0.002)
**IMH**	37 (5.8)	32 (5.0)	0.180	0.008 (−0.003–0.013)
**PAU**	37 (5.8)	30 (4.7)	0.065	0.011 (−0.001–0.016)

Data are shown as number (percentage).

Difference = Contrast-only − Full protocol.

Differences between protocols were assessed using the McNemar’s exact test.

IMH, intramural hematoma; PAU, penetrating aortic ulcer; CI, confidence interval.

**Table 3:** Diagnostic performance of the contrast-only protocol for major aortic pathologies using the full protocol as the reference standard.

Pathology	TN	FP	FN	TP	Sensitivity (95% CI)	Specificity (95% CI)
**Aneurysm**	425	0	0	215	100% (98.2–100)	100% (99.1–100)
**Dissection**	538	0	2	100	98.0% (93.1–99.5)	100% (99.3–100)
**IMH**	601	7	2	30	93.8% (79.9–98.3)	98.8% (97.6–99.4)
**PAU**	601	9	2	28	93.3% (78.7–98.2)	98.5% (97.2–99.2)

TN = true negative; FP = false positive; FN = false negative; TP = true positive. Values in parentheses indicate 95% confidence intervals (Wilson method). The full protocol was used as the reference standard. IMH = intramural hematoma; PAU = penetrating atherosclerotic ulcer.

**Fig 1:**
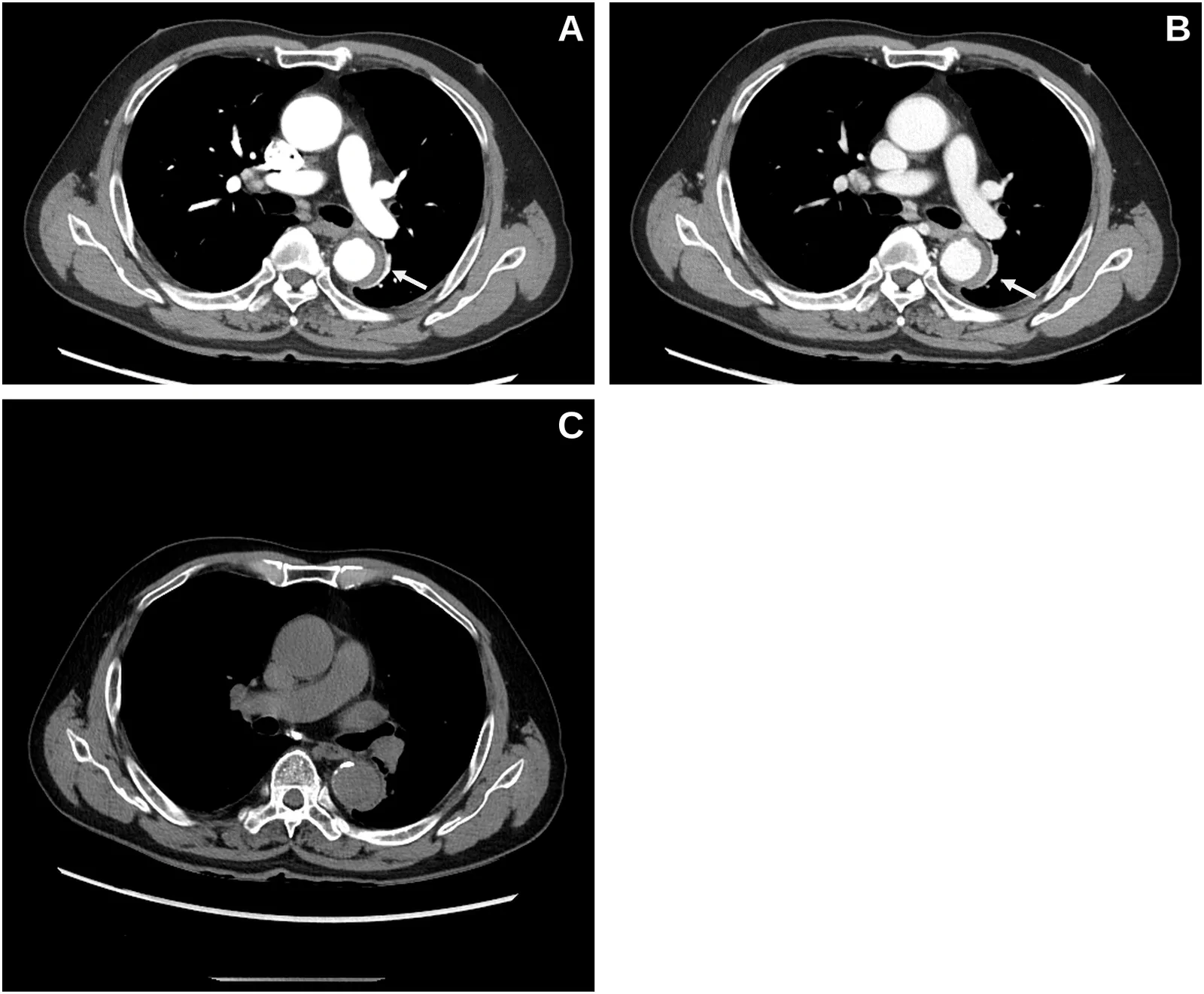
An 81-year-old man presenting with chest pain. (A, B) Contrast-enhanced CT angiography in the arterial (A) and delayed (B) phases demonstrates a crescent-shaped high-attenuation mural lesion along the descending thoracic aorta (arrow), consistent with intramural hematoma (IMH). The lesion shows no enhancement on delayed imaging, supporting its non-enhancing nature. (C) Unenhanced CT image confirms corresponding high attenuation within the aortic wall (arrow), consistent with acute intramural hematoma.

**Fig 2:**
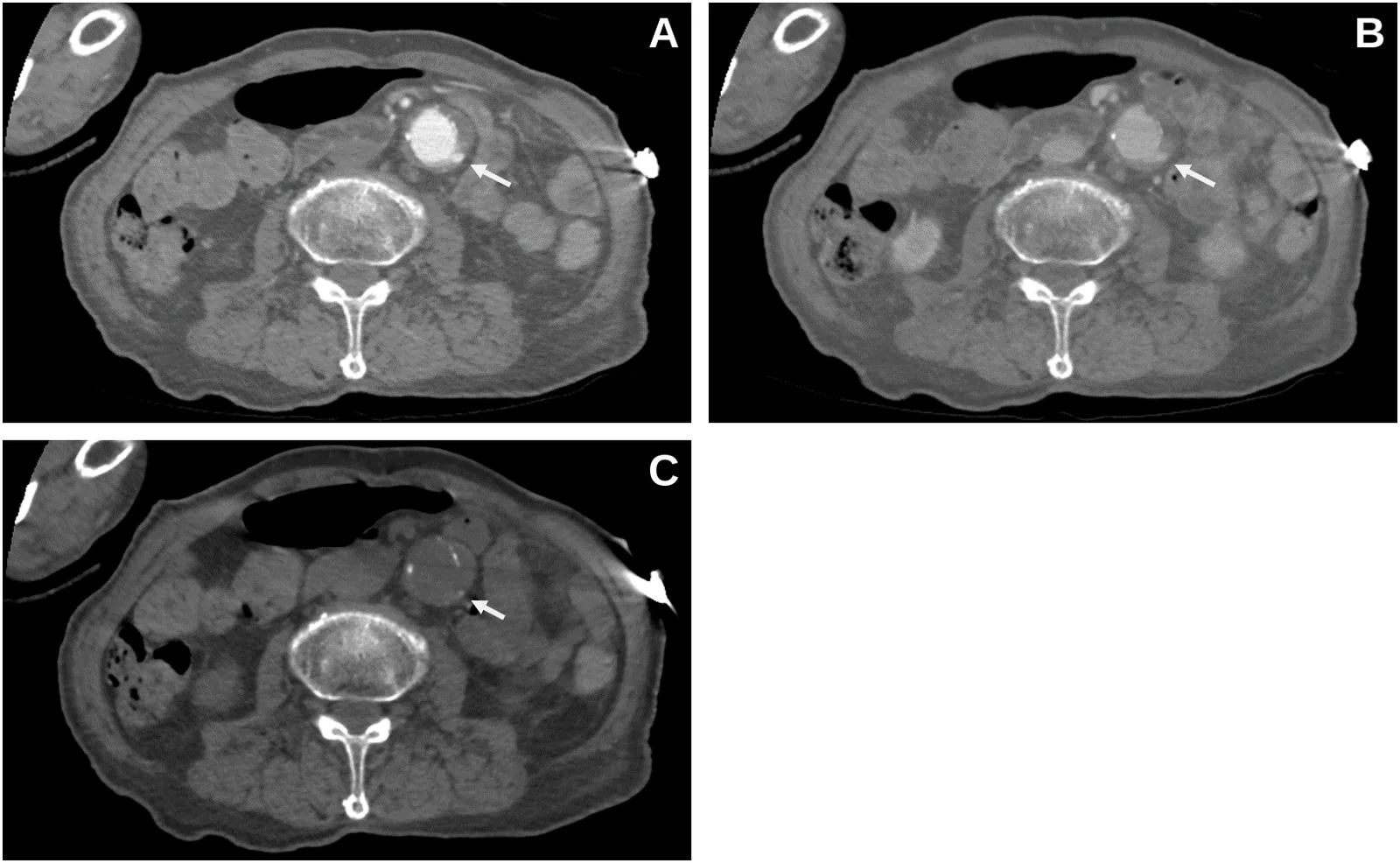
An 83-year-old man undergoing CT angiography. (A, B) Contrast-enhanced CT angiography in the arterial (A) and delayed (B) phases demonstrates a focal contrast-filled outpouching along the aortic wall (arrow), initially suggestive of a penetrating atherosclerotic ulcer (PAU). (C) Unenhanced CT image reveals that the corresponding lesion represents intimal calcification (arrow), rather than true contrast enhancement. The calcification appears relatively indistinct on contrast-enhanced images, likely due to partial volume effects and surrounding contrast opacification, leading to false-positive interpretation.

For characterization of aneurysm, contrast-only protocol and full protocol shows substantial to perfect interprotocol agreement, with kappa values ranging from 0.703 to 1.000 (Table 4). Macroscopic aneurysm shape demonstrated perfect agreement, with identical classification of saccular (n = 29, 13.5%) and fusiform aneurysms (n = 186, 86.5%) in both protocols (κ = 1.000). Rare but clinically important findings such as the draped aorta sign and aneurysm rupture also showed perfect agreement, with identical detection across protocols (κ = 1.000 for both features, Fig 3). Assessment of mural thrombus showed substantial interprotocol agreement (κ = 0.703; 95% CI, 0.623–0.788, Fig 4). Although absolute frequencies differed slightly between protocols; absence of thrombus (58.1% vs. 47.9%), homogeneous thrombus (34.9% vs. 39.1%), crescent-shaped thrombus (6.0% vs. 10.2%), and hemorrhage into thrombus (0.9% vs. 2.8%), overall categorical concordance remained acceptable. Evaluation of intimal calcification demonstrated near perfect agreement between protocols (κ = 0.853; 95% CI, 0.751–0.932), with nearly identical distributions of absent calcification (17.7% vs. 16.3%), calcification without tangential calcium sign (80.5% vs. 81.4%), and calcification with tangential calcium sign (1.9% vs. 2.3%).

**Table 4:** Characterization of aneurysm (n = 215).

Feature	Category	Contrast-only, n (%)	Full protocol, n (%)	κ (95% CI)
**Macroscopic shape**	Saccular	29 (13.5)	29 (13.5)	1.000 (1.000-1.000)
	Fusiform	186 (86.5)	186 (86.5)	
**Mural thrombus**	Absent	125 (58.1)	103 (47.9)	0.703 (0.623–0.788)
	Homogeneous	75 (34.9)	84 (39.1)	
	Crescent-shaped	13 (6.0)	22 (10.2)	
	Hemorrhage	2 (0.9)	6 (2.8)	
**Intimal calcification**	Absent	38 (17.7)	35 (16.3)	0.853 (0.751–0.932)
	Present w/o tangential sign	173 (80.5)	175 (81.4)	
	Present with tangential sign	4 (1.9)	5 (2.3)	
**Draped aorta sign**	Absent	211 (98.1)	211 (98.1)	1.000 (1.000-1.000)
	Present	4 (1.9)	4 (1.9)	
**Ruptured aneurysm**	Absent	210 (97.7)	210 (97.7)	1.000 (1.000-1.000)
	Present	5 (2.3)	5 (2.3)	

Data are shown as number (percentage). Interprotocol agreement was assessed using Cohen’s kappa (κ) with 95% confidence intervals.

IMH, intramural hematoma; PAU, penetrating aortic ulcer; CI, confidence interval.

**Fig 3:**
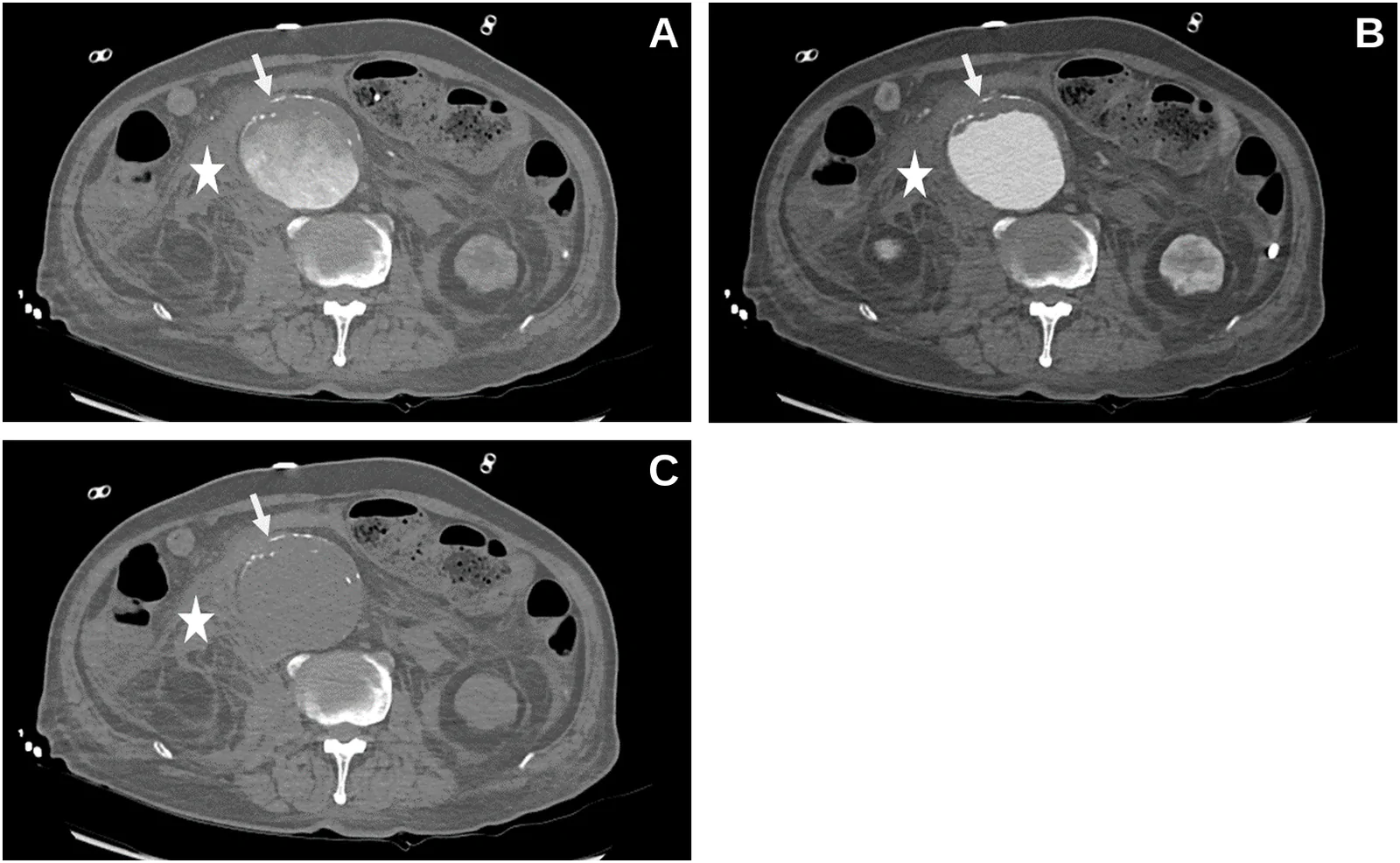
An 87-year-old man presenting with abdominal pain. (A, B) Contrast-enhanced CT angiography in the arterial (A) and delayed (B) phases demonstrates a markedly dilated abdominal aortic aneurysm measuring 7.4 cm in diameter. Focal outward displacement of intimal calcification (arrow), consistent with the tangential calcium sign, is noted along the aneurysmal wall, suggesting impending rupture. A surrounding retroperitoneal hematoma (asterisk) is also present, confirming associated aneurysm rupture. (C) Unenhanced CT image demonstrates the same displaced intimal calcification (arrow) and corresponding mural abnormality, indicating that the key diagnostic features are identifiable even without contrast administration.

**Fig 4:**
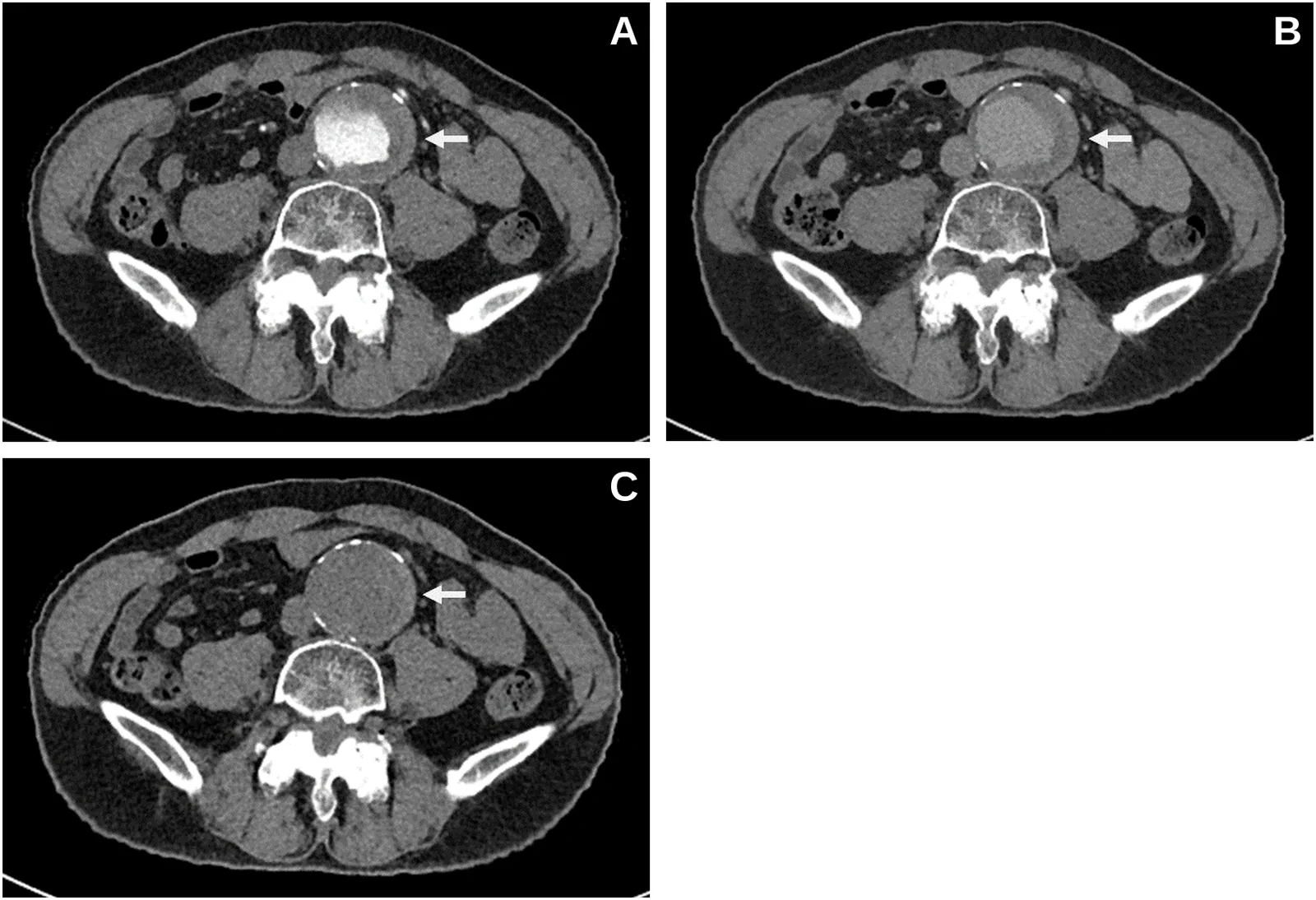
A 68-year-old man presenting with abdominal pain. (A, B) Contrast-enhanced CT angiography in the arterial (A) and delayed (B) phases demonstrates a 5-cm abdominal aortic aneurysm. A crescent-shaped, high-attenuating area is seen with intimal calcification along the outer aortic wall. This finding is consistent with a high-attenuating mural thrombus, indicating an impending risk of rupture. (C) Unenhanced CT image demonstrates the same high-attenuation crescent within the mural thrombus (arrow), confirming the finding.

### Agreement analysis

Both inter-reader agreement and interprotocol agreement were assessed. Detailed results for detection of aortic pathologies are presented in Table 5, and results for aneurysm characterization are presented in S3 Table.

**Table 5:** Interprotocol and inter-reader agreement for detection of major aortic pathologies.

Pathology	Agreement type	Protocol/ Reader	Κ (95% CI)	Δκ	p-value
**Aneurysm**	Interprotocol	R1	1.000 (1.000–1.000)		
		R2	1.000 (1.000–1.000)		
	Inter-reader	Contrast-only	0.787 (0.736–0.834)	0.000 (0.000-0.000)	1.000
		Full protocol	0.787 (0.730–0.834)		
**Dissection**	Interprotocol	R1	0.994 (0.981–1.000)		
		R2	0.976 (0.950–0.995)		
	Inter-reader	Contrast-only	0.970 (0.943–0.994)	0.005 (−0.002-0.012)	0.250
		Full protocol	0.988 (0.969–1.000)		
**IMH**	Interprotocol	R1	0.840 (0.735–0.924)		
		R2	0.849 (0.765–0.923)		
	Inter-reader	Contrast-only	0.761 (0.638–0.860)	0.000 (−0.011-0.011)	1.000
		Full protocol	0.776 (0.662–0.876)		
**PAU**	Interprotocol	R1	0.819 (0.701–0.919)		
		R2	0.812 (0.657–0.928)		
	Inter-reader	Contrast-only	0.698 (0.532–0.832)	0.006 (−0.004-0.017)	0.260
		Full protocol	0.768 (0.620–0.890)		

Note. Agreement was assessed using Cohen’s kappa (κ) with 95% confidence intervals. Δκ denotes the difference in inter-reader agreement between the full and contrast-only protocols.

IMH, intramural hematoma; PAU, penetrating aortic ulcer; CI, confidence interval.

#### Agreement for detection of aortic pathologies.

Interprotocol agreement between the contrast-only and full protocols was excellent across all aortic pathologies in both readers (Table 5). Both readers demonstrated perfect agreement for aneurysm detection (κ = 1.000 for R1 and R2). Dissection also showed near-perfect concordance (κ = 0.994 for R1; κ = 0.976 for R2), with narrow confidence intervals. Agreement for IMH (κ = 0.840 for R1; κ = 0.849 for R2) and PAU (κ = 0.819 for R1; κ = 0.812 for R2) remained near excellent. Inter-reader agreement similarly demonstrated high reliability for all pathologies in both protocols. For aneurysm detection, kappa values were identical between contrast-only and full protocols (κ = 0.787 for both; *p* = 1.000). Dissection also showed excellent consistency between readers (κ = 0.970 vs. 0.988), without a statistically significant interprotocol difference (*p* = 0.25). Agreement for IMH (κ = 0.761 vs. 0.776) and PAU (κ = 0.698 vs. 0.768) showed no significant differences between protocols (*p* = 1 and 0.26, respectively). Collectively, these findings indicate that reader agreement with the contrast-only protocol was comparable to that of the full protocol.

#### Agreement for characterization of aneurysm.

Interprotocol agreement based on individual reader assessments also showed substantial to perfect agreement for most features, with kappa values ranging from 0.972 to 1.000 (S3 Table). Thrombus characterization demonstrated relatively lower agreement, with moderate to substantial concordance (κ = 0.644 for reader 1 and κ = 0.563 for reader 2). Inter-reader agreement was similarly robust across aneurysm features for both protocols. Macroscopic shape showed identical inter-reader agreement for contrast-only and full protocols (κ = 0.644 for both), while thrombus evaluation demonstrated moderate to substantial agreement (κ = 0.667 and 0.738). Calcification showed substantial to excellent concordance (κ = 0.814 and 0.796). For the draped aorta sign, inter-reader agreement was near-perfect, although influenced by its low prevalence. Rupture showed perfect inter-reader agreement across both protocols (κ = 1.000).

## Discussion

In this study, we evaluated whether a contrast-only CTA protocol could provide diagnostic performance and reader reliability comparable to those of a conventional multiphase CTA protocol in patients with confirmed acute aortic pathology. Our results demonstrate that the contrast-only protocol was non-inferior to the full protocol for the detection of major aortic diseases, including aneurysm, dissection, IMH, and PAU, while achieving a meaningful reduction in radiation exposure. Aneurysm characterization and inter-reader agreement were similarly preserved. However, as discussed further below, important limitations of the study design—particularly the inclusion of only pathology-confirmed cases—should be carefully considered when interpreting these findings.

For the detection of aortic aneurysms and dissections, the contrast-only protocol showed near-perfect concordance with the full protocol. The detection rates for aneurysms were identical between the two protocols. The marginal difference in detecting dissection was contributed from two discordant cases, both representing false-positive interpretations on contrast-only imaging. One case corresponded to a penetrating atherosclerotic ulcer that was misinterpreted as a focal dissection, likely due to the pseudo-flap appearance created by ulceration and adjacent calcification. The other case involved a focal intimal flap with calcification, which was subsequently considered a chronic post-dissection change rather than an active dissection on review of unenhanced images. Importantly, neither case represented a clinically actionable acute dissection, and therefore these discrepancies were unlikely to have impacted patient management.

While unenhanced imaging is traditionally deemed essential for diagnosing IMH and differentiating mural hyperattenuation from contrast enhancement, our study demonstrated the detection rates of IMH and PAU did not differ significantly between the contrast-only protocol and full protocol and non-inferiority of the contrast-only protocol for IMH and PAU detection within the prespecified margin [1,4,5]. Nevertheless, contrast-only protocol showed a tendency toward higher detection rates for IMH and PAU, primarily attributable to false-positive interpretations. Without unenhanced images, calcifications, complex atherosclerotic changes, mural thrombi, or irregular aortic wall thickening may mimic acute IMH or PAU on contrast-enhanced images alone. If IMH or PAU cannot be confidently distinguished from calcification, mural thrombus, or chronic atherosclerotic change, additional unenhanced imaging should be considered. Furthermore, these false-positive findings should not be considered clinically negligible solely on the basis of statistical non-inferiority, as misdiagnosis of chronic wall abnormalities or calcification as acute IMH or PAU could lead to unnecessary hospital admission, aggressive blood pressure management, additional imaging, or other unwarranted interventions. Therefore, when the unenhanced phase is omitted, radiologists should carefully assess lesion morphology and attenuation characteristics across the available contrast-enhanced phases and compare the findings with prior examinations whenever available. Accordingly, this approach should be applied selectively, particularly in patients with known aortic disease undergoing serial surveillance, rather than as a universal replacement for conventional multiphase CTA. In terms of detailed aneurysm characterization, the contrast-only protocol proved highly reliable for most morphological assessments. Clinically important features—including macroscopic shape (saccular vs. fusiform), the draped aorta sign, and the presence of rupture—showed perfect interprotocol agreement (κ = 1.000). Intimal calcification also demonstrated near-perfect concordance (κ = 0.853), indicating that omission of the unenhanced phase does not compromise the evaluation of overall morphology, wall instability, or signs of impending rupture. In contrast, the assessment of mural thrombus showed comparatively lower, although still substantial, agreement (κ = 0.703). The contrast-only protocol tended to underestimate crescent-shaped thrombi (6.0% vs. 10.2%) and hemorrhage within the thrombus (0.9% vs. 2.8%), while overestimating the absence of thrombus (58.1% vs. 47.9%). This pattern likely reflects the inherent limitations of CT imaging in differentiating subtle intra-thrombus components. As previously reported, although CTA provides excellent accuracy for macroscopic geometric evaluation of aortic aneurysms, its ability to distinguish fine thrombus composition—such as differentiating fresh hemorrhage from organized thrombus—is limited compared with MRI due to inferior soft-tissue contrast [21]. In the present study, omission of the unenhanced phase further removed the baseline high-attenuation reference needed to identify subtle hemorrhagic components within the thrombus, which may explain the higher proportion of cases categorized as “absent” or indeterminate on contrast-only imaging. In addition, prior studies have suggested that thrombus morphology and internal composition are inherently more subjective features, contributing to increased interobserver variability [22,23]. Nevertheless, given that the most clinically critical findings—such as rupture and high-risk aneurysm morphology—were consistently identified across both protocols, the contrast-only approach remains a clinically feasible and effective imaging strategy.

Beyond diagnostic consistency, the contrast-only protocol was associated with a meaningful reduction in radiation dose (mean DLP reduction: 32.1%). This dose benefit may be especially relevant in patients requiring serial CT examinations for aortic surveillance—such as those with known aneurysms or post-interventional follow-up—where minimizing cumulative exposure across multiple examinations is an important clinical objective. The current findings provide preliminary evidence supporting the feasibility of contrast-only protocols in this specific context, though further prospective studies in broader and unselected populations are warranted before more widespread implementation.

This study has several limitations. First, it is a retrospective single-center study involving 160 patients. Although this cohort was comparable to or larger than those of several previous studies evaluating reduced-phase or optimized aortic CT protocols, the numbers of patients with relatively uncommon pathologies, particularly IMH and PAU, remained limited. Larger multicenter studies are warranted to improve the precision and generalizability of these findings. Second, and most importantly, only patients with confirmed aortic pathology were included, creating an intentionally enriched cohort. This design does not reflect the real-world emergency setting, where CTA is primarily used to exclude acute aortic syndrome in a low-to-intermediate prevalence population. The absence of true-negative cases means that our sensitivity and agreement metrics are likely higher than would be observed in unselected populations, and the study is not designed to address the question of whether the unenhanced phase can be safely omitted when ruling out acute aortic syndrome. Rather, our findings are most applicable to the context of patients with known or previously confirmed pathology undergoing serial follow-up imaging, where the diagnostic question is monitoring of established disease rather than initial exclusion. Third, the full multiphase protocol served as the reference standard, as no independent external confirmation such as surgical findings or follow-up imaging was systematically available for all included cases. This circular design is a recognized methodological limitation. Fourth, all CT examinations were performed using recent-generation CT scanners (e.g., SOMATOM Force, Revolution CT). Therefore, our findings regarding the high diagnostic yield of the arterial phase may not be fully generalizable to institutions utilizing older-generation CT equipment with inferior spatial or temporal resolution. Fifth, all image interpretations were performed by two radiologists with more than 15 years of CTA experience. Therefore, our findings may not be directly generalizable to less-experienced radiologists, particularly for subtle aortic wall abnormalities such as IMH and PAU. Further studies involving multiple readers with varying levels of experience are warranted.

## Conclusion

In conclusion, in patients with confirmed aortic pathology, a contrast-only CTA protocol demonstrated non-inferior diagnostic performance and comparable reader agreement relative to conventional multiphase CT, while achieving a meaningful reduction in radiation dose. However, given the enriched study cohort and the absence of true-negative cases, these findings should not be extrapolated to initial emergency evaluation where ruling out acute aortic syndrome is the primary goal. Rather, the results support the feasibility of a contrast-only approach in selected patients—particularly those undergoing serial surveillance imaging—as a strategy to reduce cumulative radiation exposure without compromising the characterization of established aortic pathology. Prospective validation in broader, unselected populations is needed before wider clinical adoption.

## Supporting information

S1 Table CT acquisition parameters according to CT scanner. (DOCX)

S2 Table CT definition of characterization of aneurysm morphology. (DOCX)

S3 Table Interprotocol and inter-reader agreement for characterization of aneurysm. (DOCX)

S4 Table Sensitivity analysis of detection differences using generalized estimating equation models. (DOCX)
