# Supplementary material for: Feasibility of omitting the unenhanced phase in CT angiography for acute aortic pathologies
Source: PLoS One. 2026 Nov 13;21(11):e0362760. doi: 10.1371/journal.pone.0362760 (PMC13766274; doi:10.1371/journal.pone.0362760)
Supplement: Supplementary file 1 [file pone.0362760.s001.docx]

**S1 Table. CT acquisition parameters according to CT scanner**

| Parameter | SOMATOM Force (Siemens Healthineers) | Revolution Frontier CT / Revolution CT (GE Healthcare) |
| --- | --- | --- |
| Tube voltage (kVp) | Automated kVp selection (CARE kV), reference 100–120 kVp | Automated kVp selection (kV Assist), reference 100–120 kVp |
| Tube current | Automated tube current modulation (CARE Dose4D) | Automated tube current modulation (Smart mA) |
| Rotation time (s) | 0.25–0.28 | 0.35–0.50 |
| Pitch | 0.6–1.2 (adaptive) | 0.98–1.375 |
| Slice thickness (reconstruction) | 2.0 mm | 2.0 mm |
| Reconstruction interval | 2.0 mm | 2.0 mm |
| Reconstruction algorithm | ADMIRE (strength 2–3) | ASiR-V (40–60%) |
