# Supplementary material for: Feasibility of omitting the unenhanced phase in CT angiography for acute aortic pathologies
Source: PLoS One. 2026 Nov 13;21(11):e0362760. doi: 10.1371/journal.pone.0362760 (PMC13766274; doi:10.1371/journal.pone.0362760)
Supplement: Supplementary file 2 [file pone.0362760.s002.docx]

**S2 Table. CT definition of characterization of aneurysm morphology**

| Feature | Category | CT Definition |
| --- | --- | --- |
| Macroscopic shape | Saccular | Asymmetric, localized outpouching involving a portion of the aortic circumference. |
|  | Fusiform | Symmetric, circumferential dilatation involving the entire aortic circumference. |
| Mural thrombus | Absent | No intraluminal thrombus along the aneurysm wall. |
|  | Homogeneous | Uniform low-attenuation thrombus with smooth inner margin. |
|  | Crescent-shaped | Eccentric, crescentic thrombus with nonuniform thickness. |
|  | Hemorrhage into thrombus | Areas of increased attenuation within thrombus suggesting acute or subacute hemorrhage. |
| Intimal calcification | Absent | No visible calcification along the aneurysm wall. |
|  | Present without tangential calcium sign | Calcification present but without displacement or stretching along the outer wall. |
|  | Present with tangential calcium sign | Curvilinear calcification displaced toward the outer contour of the aneurysm, suggestive of wall instability. |
| Draped aorta sign | Absent | Preserved fat plane between the posterior aortic wall and adjacent vertebral body. |
|  | Present | Loss of posterior aortic wall convexity with close apposition or “draping” of the aorta over the vertebral body. |
| Rupture | Absent | No evidence of periaortic hematoma, contrast extravasation, or retroperitoneal hemorrhage. |
|  | Present | Presence of periaortic hematoma, active contrast extravasation, or contained rupture. |
