# Supplementary material for: Feasibility of omitting the unenhanced phase in CT angiography for acute aortic pathologies
Source: PLoS One. 2026 Nov 13;21(11):e0362760. doi: 10.1371/journal.pone.0362760 (PMC13766274; doi:10.1371/journal.pone.0362760)
Supplement: Supplementary file 3 [file pone.0362760.s003.docx]

**S3 Table. Interprotocol and inter-reader agreement for characterization of aneurysm**

| Feature | Agreement type | Reader / Protocol | Κ (95% CI) |
| --- | --- | --- | --- |
| Macroscopic shape | Interprotocol | R1 | 1.000 (1.000–1.000) |
|  |  | R2 | 1.000 (1.000–1.000) |
|  | Inter-reader | Contrast-only | 0.644 (0.398–0.834) |
|  |  | Full protocol | 0.644 (0.410–0.824) |
| Mural thrombus | Interprotocol | R1 | 0.644 (0.546–0.736) |
|  |  | R2 | 0.563 (0.467–0.658) |
|  | Inter-reader | Contrast-only | 0.667 (0.566–0.757) |
|  |  | Full protocol | 0.738 (0.657–0.814) |
| Intimal calcification | Interprotocol | R1 | 0.927 (0.840–0.985) |
|  |  | R2 | 0.952 (0.887–1.000) |
|  | Inter-reader | Contrast-only | 0.814 (0.696–0.912) |
|  |  | Full protocol | 0.796 (0.677–0.903) |
| Draped aorta sign | Interprotocol | R1 | 0.797 (0.000–1.000) |
|  |  | R2 | 1.000 (1.000–1.000) |
|  | Inter-reader | Contrast-only | −0.007 (−0.016–0.000) |
|  |  | Full protocol | −0.008 (−0.019–0.000) |
| Ruptured aneurysm | Interprotocol | R1 | 1.000 (1.000–1.000) |
|  |  | R2 | 1.000 (1.000–1.000) |
|  | Inter-reader | Contrast-only | 1.000 (1.000–1.000) |
|  |  | Full protocol | 1.000 (1.000–1.000) |

**Note.** Agreement was assessed using Cohen’s kappa (κ) with 95% confidence intervals. Negative κ values reflect very low prevalence of the corresponding finding

κ, Cohen’s kappa; CI, confidence interval
