# Supplementary material for: Feasibility of omitting the unenhanced phase in CT angiography for acute aortic pathologies
Source: PLoS One. 2026 Nov 13;21(11):e0362760. doi: 10.1371/journal.pone.0362760 (PMC13766274; doi:10.1371/journal.pone.0362760)
Supplement: Supplementary file 4 [file pone.0362760.s004.docx]

**S4 Table.** **Sensitivity analysis of detection differences using generalized estimating equation models**

| **Pathology** | **Difference, 95% CI** | **Within non-inferiority margin (**−**5%)** |
| --- | --- | --- |
| Aneurysm | 0.000 (0.000, 0.000) | Yes |
| Dissection | −0.003 (−0.007, 0.001) | Yes |
| IMH | 0.008 (−0.001, 0.017) | Yes |
| PAU | 0.011 (0.002, 0.020) | Yes |

Differences in detection proportions between the contrast-only and full protocols were estimated using generalized estimating equation models.

The prespecified non-inferiority margin was 5% (Δ = −0.05).

Non-inferiority was established when the lower bound of the 95% CI was greater than −0.05.

IMH, intramural hematoma; PAU, penetrating aortic ulcer; CI, confidence interval.
